# Barriers and facilitators to high-volume evidence-based innovation and implementation in a large, community-based learning health system

**DOI:** 10.1186/s12913-024-11803-5

**Published:** 2024-11-21

**Authors:** Cimone Durojaiye, Stephanie Prausnitz, Jennifer L. Schneider, Tracy A. Lieu, Julie A. Schmittdiel, Smita Rouillard, Yi-Fen Chen, Kristine Lee, Douglas A. Corley

**Affiliations:** 1 Division of Research, Kaiser Permanente Northern California, Pleasanton, CA USA; 2 The Permanente Medical Group, Pleasanton, CA USA

**Keywords:** Implementation, CFIR, Learning health system, Barriers, Facilitators

## Abstract

**Background:**

Broad-scale, rapid health care change is critically needed to improve value-based, effective health care. Health care providers and systems need to address common barriers and facilitators across the evidence to implementation pathway, across diverse specialties. However, most evidence translation / implementation research evaluates single topic areas, and may be of limited value for informing comprehensive efforts. This project’s objective was to identify, characterize, and illustrate common trans-topic facilitators and barriers of translating new health care evidence results to clinical implementation across multiple medical specialties.

**Methods:**

This study was an evaluation of all evidence-based innovation projects completed during 2019–2021. Each project was created with medical group clinical leaders and was intended to inform clinical care. The evaluation took place in a large community-based integrated health care system, and an embedded delivery science and applied research program. Clinical investigators, scientific investigators, and clinical operational leaders received structured questionnaires regarding barriers and facilitators for the operational implementation of new research findings for each project. Responses were mapped to the Consolidated Framework for Implementation Research to identify perceived implementation barriers and facilitators.

**Results:**

All 48 projects completed between 2019 and 2021 were evaluated; responses were received for 45 (94%) and 34 had comments mappable to framework domains. Potential barriers and facilitators to clinical implementation of new research results were identified across all five framework domains and, within these, the 38 constructs or sub-constructs. Among 245 total comments, the most commonly cited facilitators were how the new research evidence generated, compelled change (*n* = 29), specialty communication networks for disseminating results and initiating change (*n* = 20), leadership engagement in the project (*n* = 19), and the innovation’s relative advantage over existing practices (*n* = 11). The most commonly cited barriers were inadequate resource commitment for next-step implementation (*n* = 15), insufficient learning/implementation culture (*n* = 5), and insufficient individual-level willingness/ability for change (*n* = 5).

**Conclusions:**

A novel large-scale evaluation of barriers and facilitators across the evidence to implementation pathway identified common factors across multiple topic areas and specialties. These common potentially replicable facilitators and modifiable barriers can focus health systems and leaders pursuing large-volume evidence-to-implementation initiatives on those areas with the likely greatest benefit-for-effort, for accelerating health care change.

**Supplementary Information:**

The online version contains supplementary material available at 10.1186/s12913-024-11803-5.

## Background

The expeditious translation of evidence into clinical practice poses a substantial challenge to the United States health care system [1]. Broad-scale, rapid health care change is critical for improving value-based, effective health care and for enabling the evidence-to-implementation-to-evaluation continuum for large numbers of projects across multiple disciplines [2]. However, creating the infrastructure and systems to address this need, conceptualized by the Institute of Medicine’s seminal evidence-based medicine workshop, requires understanding which facilitators and barriers commonly impact this continuum across diverse health topics, specialties, and settings [1, 3]. National trends toward more integrated healthcare systems, including the development of Accountable Care Organizations, offer great potential for achieving this goal [4]. However, few studies of common facilitators and barriers of the evidence-to-implementation continuum across many topic types exist to inform such cross-cutting systems for high-volume, rapid change.

Most in-depth implementation research is topic-based rather than system-based across multiple topics. Implementation research seeks to “adopt and integrate evidence-based health interventions into clinical and community settings for the improvement of patient outcomes and patient/provider experiences that benefit population health” [5]. Yet, traditional implementation studies usually provide an in-depth focus on a single subject in a specific setting or specialty [6–10]. While useful for the question of interest, single-topic studies may not identify systemic issues [11]. In contrast, evaluating themes across multiple projects, specialties, and medical centers can inform potentially replicable and generalizable changes across a learning health system.

To address these evidence gaps, we evaluated barriers and facilitators to implementation across the research-to-implementation continuum among numerous, diverse projects within a delivery science and applied research program in a large, multi-center, community-based health system, using the Consolidated Framework for Implementation Research (CFIR).

## Methods

### Setting and population

This evaluation was conducted within The Permanente Medical Group’s Delivery Science and Applied Research (DARE) program at Kaiser Permanente Northern California, an integrated health care system with approximately 4.6 million members, 9500 physicians, and 21 medical centers [12, 13]. The setting provides care for a racially, ethnically, and socioeconomically diverse population which closely reflects the region’s underlying census population, including by insurance type (commercial, Medicare, etc.) [14, 15]. 

The DARE program [16] provides personnel and funding to support clinicians in answering actionable, high priority questions to address evidence gaps and inform evidence-based changes in clinical care across all medical and surgical specialties. Each project is a collaboration between a clinical and a scientific co-principal investigator. Projects are identified, developed, and completed in consultation with medical group executives who lead clinical operations. The program utilizes evidence-based implementation methods [17] throughout each project’s cycle.

### Project survey targets and content

**Fig. 1:**
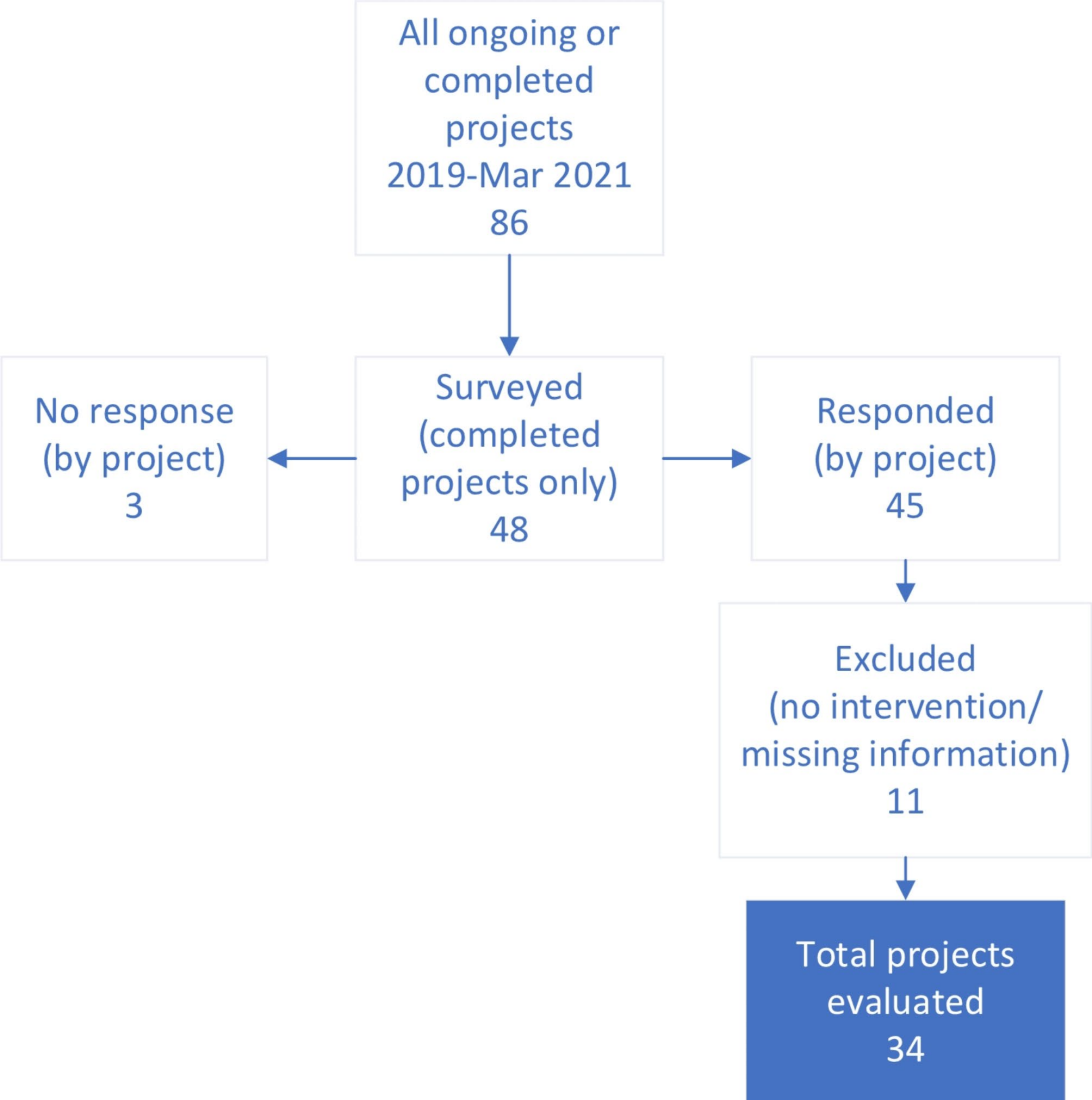
DARE projects included in the survey

During the study period of 2019–2021, there were 86 ongoing or completed DARE projects; all 48 projects completed during this period were included in the survey (Fig. 1). Surveys were fielded to each project’s lead scientific investigator and to regional operational and clinical leaders within the project’s specialty area, who were encouraged to forward it to other relevant leaders (Table 1). The goal was to understand the end-user’s knowledge of the project’s findings and to identify perceived barriers and facilitators to implementation by both project team leaders and the specialty’s end-users (i.e., clinical leaders). For each project, responses were considered “received” if there was at least one response for the project. Received responses were included for analyses if the results indicated a logical next step for implementation and if there were specific comments about implementation barriers or facilitators that were mappable to the framework domains. Depending upon the project, the survey could have been completed pre-implementation of any contemplated changes, during implementation, or post-implementation. The phase at survey was determined in part by whether the project proceeded successfully to implementation.

**Table 1:** Participant characteristics

	^a^*n* (%)	^b^*n* (%)
Participant’s Clinical Title		
Clinician Investigator	42 (51.9)	32 (47.8)
Regional Chair of Chiefs	21 (25.9)	17 (25.4)
Associate Executive Director	1 (1.2)	1 (1.5)
Other	3 (3.7)	3 (4.5)
Dual (Clinician Investigator/Clinical Leader)	5 (6.2)	5 (7.5)
No response	9 (11.1)	9 (13.4)
**Total**	**81**	**67**

^a^Survey respondents for eligible projects (45 projects were eligible for evaluation)

^b^Survey respondents for evaluated projects (34 projects were evaluated)

Potential participants received an emailed survey with fifteen structured quantitative and qualitative questions regarding the study’s perceived effectiveness, the dissemination of research findings, and facilitators or barriers that impacted potential operational implementation of findings for clinical change (Appendix A). Questions utilized plain language to minimize unfamiliar jargon and had separate queries for implementation facilitators and barriers. They incorporated both quantitative (using Likert scales) and open-ended response questions unconstrained, given the clinical audience, by unfamiliar constructs or terminology. Survey domains included: dissemination practices; target audiences and communication methods; specific clinical or operational changes informed by the project’s results; facilitators and barriers of translating the results to implementation; any additional perceived benefits of the project for the individual or specialty, such as development of investigative experience or career paths; and recommendations for program improvement, including for translating project research results to implementation.

The KPNC Research Determination Committee determined the project did not meet the regulatory definition of research involving human subjects requiring institutional board approval.

### Analysis

The analysis aimed to (1) identify perceived project-specific facilitators and barriers of research implementation, (2) map concepts from participant’s comments to the CFIR constructs, and (3) identify facilitators and barriers common across multiple projects [18]. 

CFIR is a widely accepted framework for assessing barriers and facilitators of implementation [7, 10, 19–26]. Its creation involved reviews of several hundred publications across multiple scientific disciplines followed by the combination of different constructs into a single framework [26]. The current project utilized the full core CFIR framework, without a recently proposed six-element addendum regarding anticipated vs. actual outcomes [18, 22]. The core framework includes five domains: innovation, outer setting, inner setting, individuals, and implementation process. These domains then divide into 26 constructs and, for three constructs with sub-constructs, 15 sub-constructs (Appendix B).

Participant responses were downloaded into matrices, grouped by project. Three reviewers (CD, SP, and DAC) independently assessed qualitative survey responses for each project, mapped each response to the applicable CFIR domains, constructs, and sub-constructs, and designated each response as a potential barrier or a facilitator to the relevant constructs or sub-constructs. Compound comments that included both barrier and facilitator components could have each comment element assigned separately to a different category. Consistent with qualitative analytic methods [27, 28], initial assignments were then re-reviewed for consistency and, where there was discordance, discussed for final consensus assignment (CD and SP).

We used the SQUIRE checklist when writing our report [Ogrinc G, Davies L, Goodman D, Batalden P, Davidoff F, Stevens D. SQUIRE 2.0 (Standards for QUality Improvement Reporting Excellence): revised publication guidelines from a detailed consensus process.].

## Results

Eighty-one survey responses were received across 45 of the 48 completed projects (94%); these included respondents from 21 different medical specialties. Eleven projects were excluded given no specific described next-step implementation relevant for the study’s results or because the comments lacked sufficient detail for construct mapping, resulting in 34 projects for the final analysis (Fig. 1; Table 1). Comments mapped to more than one construct or that included both facilitator and barrier attributes were counted multiple times. Among 245 total comments abstracted from analyzed projects, potential barriers or facilitators of implementation were identified across all five CFIR domains and across 32 of 38 domain constructs and/or sub-constructs., Table 2 includes selected illustrative comments, among constructs coded with ≥ 3 comments.

**Table 2:** Selected examples of participant feedback mapped to CFIR construct^a^

Domain/Construct/Subconstruct	Facilitator	Barrier
I. Innovation		
a. Evidence strength and quality	*“Dermoscopy use for teledermatology was strongly recommended as the standard of care by the specialty when primary care referring for skin lesions.”*	
b. Relative advantage	*“Acceptance of the concept of “regionalization” for cancer care*,* and acceptance that laparoscopic surgery (MIS = minimally invasive surgery) was superior to open surgery.”*	
c. Complexity		*“We need a standardized approach to coding*,* to identify patients with eating disorders and their treatment courses.”*
II. Inner Setting		
a. Networks & communications	*“Early relationships with ED and cardiology chairs; iterative process of improving the tool and giving feedback on preliminary results along the way.”*	
b. Readiness for implementation		
i. Leadership engagement	*“I think having regional leads and physicians as part of the study brought forth the value of the order set as well understanding how it was implemented.”*	
ii. Available resources		*“Need to have ongoing data support from Region to continue to implement change in practice.”*
iii. Access to knowledge & information	*“Firstly*,* having clear and undeniable data that showed the superiority of the new regionalized MIS approach vs the traditional approach to care. Frequent updates by lead (ST) to Surgery Chiefs at their meetings to continually socialize the project and to explain the “why” behind the needed changes (based on the data) to our regional organization of gastric cancer care.”*	
c. Culture		*“Some medical centers wanted to stick with their current workflow out of ease rather than effectiveness and cost-savings.”*
d. Structural characteristics		*“Communication and complexity of our very large medical group. Education and adoption are difficult without some automation to make work of implementation easier for very busy AFM docs with little time.”*
e. Implementation Climate		
i. Tension for change		*“This work examined risks of stroke with or without [surgical] intervention in a retrospective manner. Dictating practice changes based on this [observational] data … was difficult given the variability of [existing practices and beliefs between individual surgeons].”*
ii. Compatibility	*“Developed program from the ground up for population level identification of FH [familial hypercholesterolemia] and management of FH. Implemented a fully integrated pathway from patient identification to care in FH specialty Clinic in a purpose-built accountable Health Connect FH ecosystem.”*	
III. Outer Setting		
a. Peer pressure	*“The work of this paper helped lay groundwork needed to bring at least 6 different surgical service lines together to work on a specific issue that will help our Surgery Services to the next level in providing in-house complex cancer care which previously was referred out.”*	
IV. characteristics of individuals		
A. individual stage of change		*“Interpersonal conflict and competition between [clinicians] in the … group has been a huge barrier in changing regional practice to this new standard.”*
V. Process		
a. engaging		
I. key stakeholders	*“From the outset key stakeholders/thought leaders in DOR-RAU [Rapid Analytics Unit] and … [TPMG] Consulting came together to work with PI and others to define primary goals or project and develop system wide approach to identify patients and take action to address the care gap*,* with periodic meeting to monitor progress and change course as needed.”*	

^a^ Selected comments displayed for constructs mapped > 3 times

Facilitators were most commonly mapped to the inner setting (*n* = 51 comments) and the innovation characteristics (*n* = 40) domains. Barriers were reported in the inner setting (*n* = 29), individual characteristics (*n* = 5), and innovation characteristics (*n* = 4) domains.

### Innovation characteristics

#### Facilitators

**Fig. 2:**
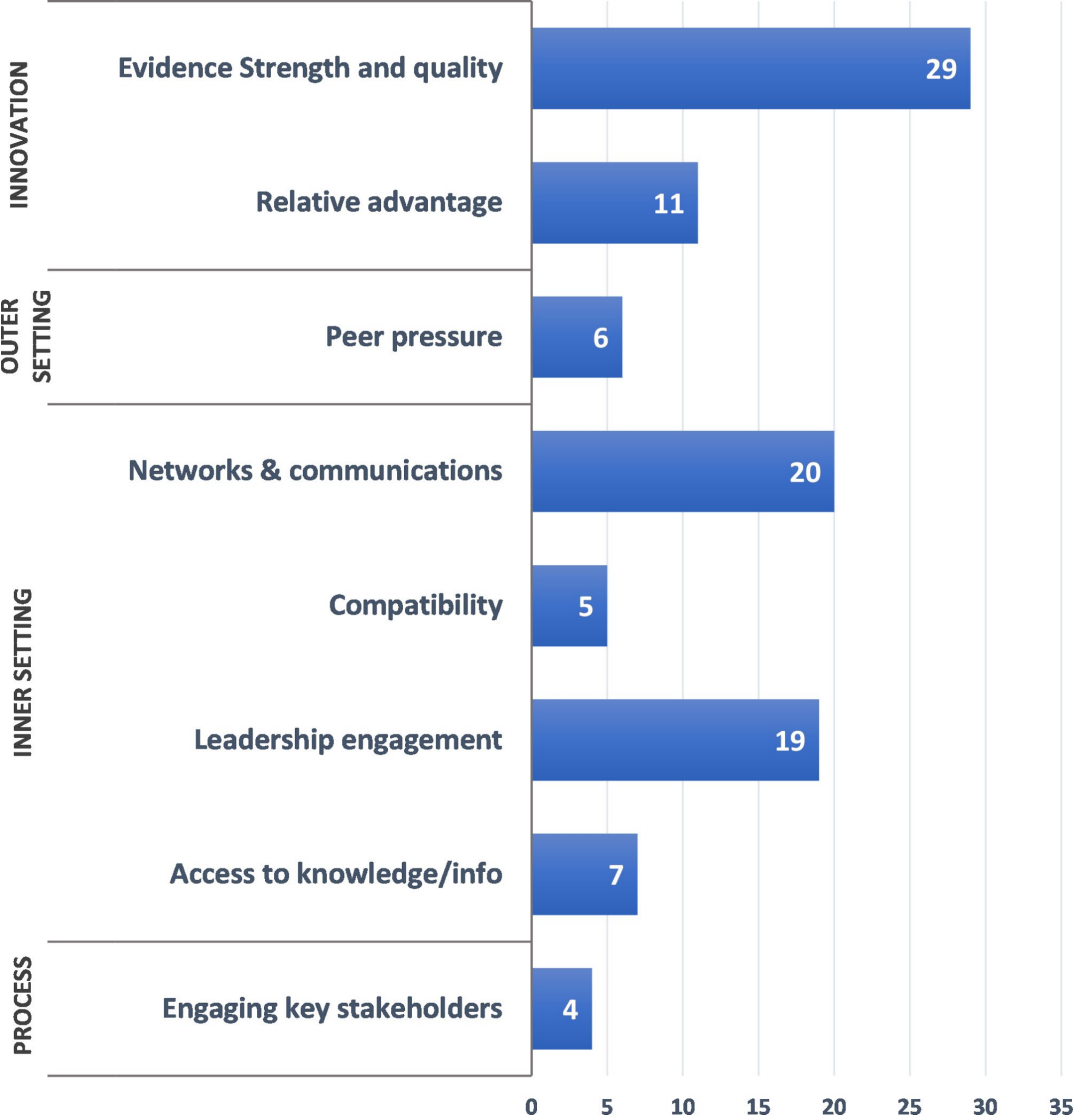
Implementation facilitators mapped to CFIR constructs^a^. Displaying constructs mapped as facilitators >3 times

The innovation domain’s *evidence strength and quality* construct was a facilitator for 29 out of 45 (64%) projects (Fig. 2). This construct reflects the stakeholder’s perception that the available evidence supports the belief that the innovation will likely achieve its desired outcomes. The *relative advantage* of the new evidence-based innovation was also frequently cited (*n* = 11). This construct incorporates the stakeholder’s perceived advantage of the studied topic vs. an alternative approach. For example, one clinician investigator noted that, for rapid delivery of clot-dissolving medications for stroke care, the project’s data supported implementation stating that, “since implementation, we have used the data collected to continue to [further] improve performance to where we are currently treating over 80% of patients in < 30 minutes, a target that was considered unachievable when we started. [These data supported] expansion to 24 × 7 and two shifts of tele-neurologists to maintain this performance.” Another project, which found a commonly performed surgical procedure to have no clear benefit, informed de-implementation, allowing “elimination of the routine placement of jejunostomy tubes pre-chemotherapy or intraoperatively [for patients undergoing certain gastrointestinal cancer surgeries]. No one in the nation was doing this yet.” Evaluation of a coordinated care team approach for patients with gastric cancer provided “clear and undeniable data that showed the superiority of the new regionalized … approach vs the traditional approach to care, and [provided] the ‘why’ behind the needed changes (based on the data) to our regional [re-] organization of gastric cancer care.” This project demonstrated this approach markedly decreased time to guideline-concordant chemotherapy, post-operative complications, and total post-operative hospital stay [29]. 

#### Barriers

**Fig. 3:**
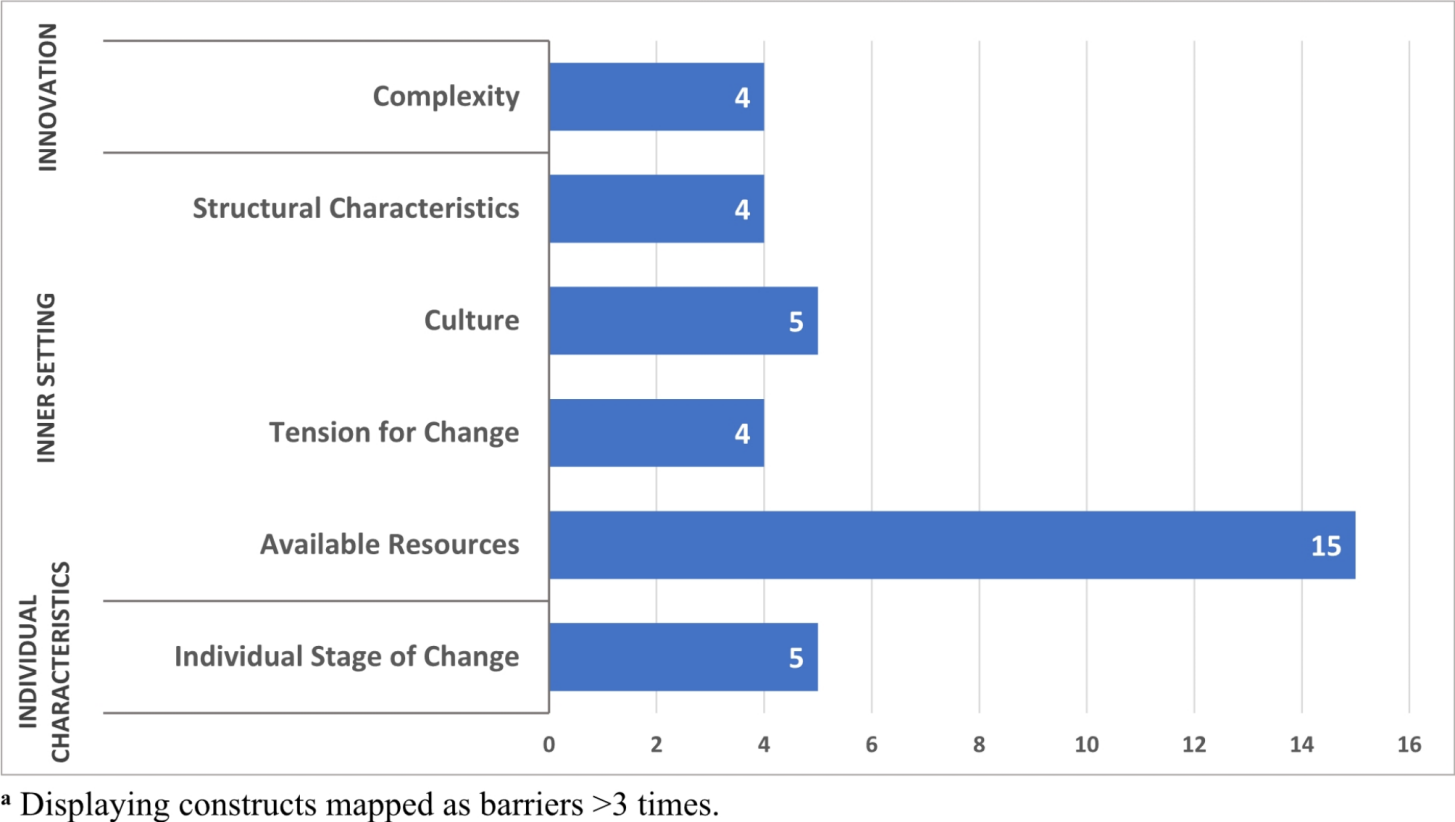
Implementation barriers mapped to CFIR constructs^a^

Within the innovation domain, innovation *complexity* was a commonly reported barrier to implementation (*n* = 4) (Fig. 3). This construct relates to the perceived difficulty for implementation of the innovation, including scope, intricacy, duration, and disruptiveness. For example, “a standardized approach to coding” was needed to identify patients with eating disorders for an intervention. However, the difficulty, accuracy, and complexity of creating such an accurate standardized electronic approach impaired broader next-step implementation.

### Inner setting

#### Facilitators

Within the inner setting domain, the *networks and communications* (*n* = 20) construct and, within the *readiness for implementation* construct, the *leadership engagement* (*n* = 19) subconstruct had the most frequent facilitator comments. The network and communications construct describes availability of developed formal and informal communications and social networks for communications within an organization. The leadership engagement subconstruct includes the perceived involvement of relevant leaders for innovation implementation. For example, a respondent for a project that evaluated the safety of a new clinical decision support tool for patients with pulmonary embolism illustrated the interplay between networks, communications, and leadership engagement: “These study results allowed us to expand tool access to non-study [emergency departments] across KPNC and teach physicians in these departments why and how the application can improve patient care… [this was facilitated by] prior relationships with the [emergency department] chiefs’ group that helped open the door for the expansion of tool access to non-study [emergency departments] across KPNC”​ [30].

#### Barriers

Several inner setting constructs were perceived barriers to implementation, particularly resources and the need for a greater learning/change culture. Within the *readiness for implementation* construct, the a*vailable resources* subconstruct, defined as sufficient dedicated organizational resources for both implementation and ongoing operations, was the most cited barrier (*n* = 15). Data support, programmers, and automation were among the most commonly resources needed. A project assessing an integrated, multidisciplinary head and neck cancer care program identified the “need to have ongoing data support from the region to continue to implement change in practice,” as a barrier to ongoing multidisciplinary tumor boards. A respondent for a study on decreased prostate cancer screening following the 2012 USPSTF guidance stated, “Education and adoption is difficult without [creating] some automation to make work of implementation easier for very busy … docs with little time; [successful implementation also needs] … a shared decision aid for screening within [the electronic medical record] and access to [medical record] programmers … so we can make changes faster.”

Within the inner setting domain’s *implementation climate* construct, several barriers were identified. Five projects cited need for an expanded *learning climate* or learning culture as a barrier, this subconstruct is related to the organization’s norms, values, and basic assumptions. One project, for example, stated that “some medical centers wanted to stick with their current workflow out of ease rather than effectiveness and cost-savings.” A project evaluating a new surgical method that would require centers of excellence stated that next-step broader implementation was “strongly opposed by the group…as compared to traditional … approaches [that were familiar to those surgeons],” reflecting the teams’ cultural hesitancy for adopting new paradigms. An additional common barrier was the *tension for change* (*n* = 4), a sub-construct reflecting stakeholders’ perceptions whether the current status warrants change. For example, the tension for change for replacing carotid stenosis surgery with medical management was insufficient despite an observational study that demonstrated comparable outcomes. Leaders stated the conservative nature of surgeons made “dictating practice changes based on this [observational] data … difficult given the variability of [existing practices and beliefs between individual surgeons].” Finally, barriers were noted within the inner setting domain’s *structural characteristics* (*n* = 4) construct, which reflect perceptions regarding the organization’s architecture, maturity, and size for implementing change. Respondents for a prostate cancer study cited, “communication and complexity of our very large medical group” as a barrier, stating that, “education and adoption is difficult without some automation to make the work of implementation easier for very busy AFM [Adult and Family Medicine] docs with little time.”

### Outer setting

#### Facilitators

Within the *outer setting* domain, the *peer pressure* construct was a commonly noted implementation facilitator (*n* = 6). This construct includes competitive pressure, where other organizations have either already implemented an innovation or there is a desire to implement it first, to gain a relative competitive advantage to the external organizations. For one care integration effort, a physician investigator noted, for example: “This effort… lays groundwork needed to bring at least 6 different surgical service lines together to work on a specific issue that will help bring our Surgery Services to the next level in providing in-house complex cancer care which previously [needed to be] referred out to [external tertiary university medical centers].” External policies and incentives were also identified as a facilitator. Two projects, one in prostate cancer screening and one regarding colorectal cancer polyp surveillance, stated that the combination of internal evidence development and relevant external care guidelines, together, compelled the implementation of practice change.

### Characteristics of individuals

#### Barriers

Within the *characteristics of individuals* domain, the *individual stage of change* (*n* = 5) construct was a commonly cited barrier to implementation. This construct refers to relevant individuals’ skilled, progressive, and sustained implementation of the evidence-based innovation. For example, one project identified “conflict and competition between [individual] surgeons in the [specific specialty surgical] group has been a huge barrier in changing regional practice to this new standard” and another project evaluating effectiveness and potential harms of a large-scale initiative changing from an inpatient to outpatient procedural workflow, identified “[clinician] reticence to change” as a key individual characteristic impeding spread and more universal implementation [31]. 

### Process

#### Facilitators

Within the *process* domain, the *engaging* construct, especially the *key stakeholder* engagement (*n* = 4) subconstruct, was the most cited facilitator to implementation. This included the investigators themselves being embedded operational leaders. For example, one project evaluating optimal surveillance strategies for hepatocellular carcinoma included regional clinical leads for the surveillance program as co-investigators; these leaders could then directly implement the evidence-based results [32]. Another project evaluating the electronic identification of patients with familial hypercholesterolemia closely incorporated both an engaged regional clinical topic-specific clinician and technological leaders for rapid deployment of a next-step population management program [33]. 

## Discussion

This study evaluated, for the first time to our knowledge, barriers and facilitators of new evidence-to-implementation cycles that are common across large numbers of specialties and project topics, using standardized data collection and a well-established conceptual framework. Across projects, the most common facilitators of implementation were the strength of the new evidence for informing clinical change, specialty communication networks for disseminating knowledge, leadership engagement, and the innovation’s relative advantage over existing practices. The main barriers were limited system-level resources (especially technological methods and personnel), need for greater embracement of a culture for learning and change, variable individual-level motivation for change, insufficient tension for change, and innovation complexity.

These findings markedly extend the current literature regarding barriers and facilitators of evidence-to-implementation continuum. To accomplish rapid-cycle, high-volume change, healthcare systems require the systematic translation of evidence-based research to practice across many topics and specialties [34]. However, most current implementation literature evaluates a single topic, a certain medical specialty setting, or, when characterizing multiple projects, may summarize disparate already-published single-topic data collected using different methods [20, 25, 35–40]. While useful for specific efforts, this approach may not identify the common and generalizable domains attainable from using a consistent, prospective approach to data collection and mapping across multiple projects. In contrast, identifying common potentially modifiable implementation facilitators and barriers across many project topics can enumerate the likely highest-yield systems-level topic areas relevant for strategic development of learning health systems [41]. The “engaging” process, for example, was identified as an important cross-topic facilitator. It includes specific efforts frequently not done, such as social marketing of the innovation, user education, training, role modeling, etc., that can accelerate the transition to implementation across multiple topics areas. Within KPNC, such strategies were effectively used by only some groups; this study’s results are now informing and replicating their broad use.

The current study has several strengths. First, it evaluated potential translation-to-implementation barriers and facilitators across many research studies using a common data collection instrument. The identification of common themes increases the likelihood that the findings are generalizable to diverse settings and topics. Second, it utilized a common framework, the Consolidated Framework for Implementation Research. CFIR’s framework can evaluate implementation before, during, or after project completion [18, 19, 24]. Thus, the results use theories, constructs, and language that are readily comparable to other settings or investigations [18, 23]. Finally, complementing studies from academic university centers, the current study’s community-based, multi- medical center setting that includes approximately 1% of people in the United States and 40% of the region’s underlying population provides common facilitators and barriers where most people receive care – community-based health care delivery systems.

Study limitations include the limited data for each project evaluated as some projects did not receive a response from multiple stakeholders. Given the large number of projects, common format for data collection, need for understandable language without jargon, and limited time availability of clinicians and operational leaders, a structured questionnaire was used rather than expansive formative or semi-structured interviews. The survey measured perceived implementation factors and did not collect data on specific implementation outcomes, thus the construct mapping does not directly evaluate successful or unsuccessful implementation beyond project leaders’ perceptions [42]. The evaluation is within a not-for-profit integrated care setting, which, while being generally comparable to how many patients in the United States are currently treated within Accountable Care Organizations, differs from some other settings [4]. The results may be less relevant for settings with multiple distinct care delivery structures between inpatient, outpatient, and specialty care or for systems with different incentive systems, such as fee-for-service; these factors may create different barriers and facilitators of implementation. The projects evaluated were also within a specific delivery science and applied research program; although the structures for innovation programs may differ, the high level of concordance across projects in this setting suggest these are likely facilitators and barriers more common to topics than to a single program. Future multi-topic evaluations in different settings will be useful to evaluate areas of concordance and discordance.

## Conclusions

In conclusion, the current analysis evaluated numerous evidence-to-implementation projects for perceived implementation facilitators and barriers, utilized an established implementation framework to classify factors across projects, and described the most common themes, with examples. These findings can inform and focus, among the large number of potential determinants described within conceptual frameworks, those areas with the likely greatest benefit-for-effort to address within multi-faceted health systems pursuing large-volume evidence-to-implementation initiatives to accelerate evidence-based care.

## Electronic supplementary material

Below is the link to the electronic supplementary material.

Supplementary Material 1.

Supplementary Material 2.

Supplementary Material 3.

## Data Availability

The datasets generated and/or analyzed during the current study are not publicly available due to the qualitative nature of the data derivation and mapping, but relevant data and approaches are available from the corresponding author on reasonable request.
