# Supplementary material for: Barriers and facilitators to high-volume evidence-based innovation and implementation in a large, community-based learning health system
Source: BMC Health Serv Res. 2024 Nov 21;24:1446. doi: 10.1186/s12913-024-11803-5 (PMC11580646; doi:10.1186/s12913-024-11803-5)
Supplement: Supplementary file 1 — Supplementary Material 1. [file 12913_2024_11803_MOESM1_ESM.docx]

**Appendix A. Delivery Science and Applied Research (DARE) Implementation Survey**

| **Survey Question** | **Response Option** |
| --- | --- |
| 1. The research project was helpful for me/our specialty for understanding or guiding clinical care for the question of interest. | - Strongly disagree - Disagree - Neutral - Agree - Strongly Agree |
| 1. Who were the study results disseminated to (choose all applicable)? | - Investigators only - Regional clinical leads - Medical center chiefs in the specialty of interest - Clinicians in the specialty of interest who treat patients with the condition/practice under study - General practice clinicians who treat patients with the condition/practice under study |
| 1. How were the results communicated? (choose all that apply) | - Email - Staff meetings - Teleconference - Other (please specify) - NA |
| 1. The project results provided information for specific changes in clinical care. | - Strongly disagree - Disagree - Neutral - Agree - Strongly Agree - NA |
| 1. What specific clinical or operational changes occurred? | - Open-ended |
| 1. Please describe any specific factors helpful for translating the study's results to changes in care (relationships, communications, connections). | - Open-ended |
| 1. Please describe any specific barriers that impeded translating the results to changes in care. | - Open-ended |
| 1. Beyond changes in care, did the study/study results indicate any next steps that should be pursued, for example, need to create better data sources or to do subsequent studies? | - Yes - No - NA |
| 1. What would the next step(s) be? | - Open-ended |
| 1. The project had other benefits/learnings that were helpful to our specialty or to other specialties. | - Strongly disagree - Disagree - Neutral - Agree - Strongly Agree |
| 1. Please describe the other benefits/learnings and how helpful. | - Open-ended |
| 1. This project was helpful for me/our specialty for developing more investigative experience for our specialty. | - Strongly disagree - Disagree - Neutral - Agree - Strongly Agree |
| 1. This project was helpful for me/our specialty in developing joy and meaning in medicine (JAMM). | - Strongly disagree - Disagree - Neutral - Agree - Strongly Agree |
| 1. Any recommendation(s) for improving this grant mechanism or for improving the DARE program’s support for you/your specialty for the transition from getting investigation results to informing implementation? | - Open-ended |
| 1. What was your role on this project? | - Clinician Investigator - Regional Chair of Chiefs - AED - Other (please specify) |
