# Supplementary material for: Barriers and facilitators to high-volume evidence-based innovation and implementation in a large, community-based learning health system
Source: BMC Health Serv Res. 2024 Nov 21;24:1446. doi: 10.1186/s12913-024-11803-5 (PMC11580646; doi:10.1186/s12913-024-11803-5)
Supplement: Supplementary file 2 — Supplementary Material 2. [file 12913_2024_11803_MOESM2_ESM.docx]

**Appendix B. Consolidated Framework for Implementation Research Codebook**

| Domain | Characteristics |
| --- | --- |
| **I. INNOVATION** | A. Innovation Source  B. Evidence Strength & Quality  C. Relative Advantage  D. Adaptability  E. Triability  F. Complexity  G. Design Quality & Packaging  H. Cost |
| **II. OUTER SETTING** | A. Needs & Resources of those the organization serves  B. Cosmopolitanism  C. Peer Pressure  D. External Policy & Incentives |
| **III. INNER SETTING** | A. Structural Characteristics  B. Networks & Communications  C. Culture  D. Implementation Climate  1. Tension for Change  2. Compatibility  3. Relative Priority  4. Organizational Incentives & Rewards  5. Goals and Feedback  6. Learning Climate  E. Readiness for Implementation  1. Leadership Engagement  2. Available Resources  3. Access to Knowledge & Information |
| **IV. CHARACTERISTICS OF**  **INDIVIDUALS** | A. Knowledge & Beliefs about the Intervention  B. Self-Efficacy  C. Individual Stage of Change  D. Individual Identification with Organization  E. Other Personal Attributes |
| **V. PROCESS** | A. Planning  B. Engaging  1. Opinion Leaders  2. Formally Appointed Internal Implementation Leaders  3. Champions  4. External Change Agents  5. Key Stakeholders  6. Innovation Participants  C. Executing  D. Reflecting & Evaluating |
