# Supplementary material for: Barriers and facilitators to high-volume evidence-based innovation and implementation in a large, community-based learning health system
Source: BMC Health Serv Res. 2024 Nov 21;24:1446. doi: 10.1186/s12913-024-11803-5 (PMC11580646; doi:10.1186/s12913-024-11803-5)
Supplement: Supplementary file 3 — Supplementary Material 3. [file 12913_2024_11803_MOESM3_ESM.docx]

**Appendix C. Evaluated Projects**

| **Project Title** |
| --- |
| A Systematic Evaluation of Eating Disorders in Children and Adolescents Identifies Patient Populations Under Care and Potential Needs |
| Chronic Anticoagulant and Antiplatelet Use Is Not Associated with Decreased Disease Severity in Sars-Cov-2 infection |
| Concomitant Cancer Treatment and SARS-Cov-Infection increased Risk of Noninvasive Ventilation Compared to Those without Cancer |
| Consolidated Multidisciplinary Care Improves Survival for Head and Neck Cancer |
| COVID-19 complications are not more common among immunosuppressed populations in KPNC |
| Decreased prostate cancer screening following the 2012 USPSTF Statement resulted in a significant increase in metastatic cancer |
| Dermoscope Use Improves Cancer Detection While Decreasing Biopsy and in Person Visits |
| Electronic Decision Support Safely Reduces Objective Cardiac Testing Among Emergency Department Patients with Chest Pain At Low Risk of Major Adverse Cardiac Events |
| Enhanced Recovery After Surgery (Eras) intervention Was Associated with Reduced Opioid Prescriptions After Surgery |
| Identifying Variation and Barriers to Use of Non-Invasive Cardiac Imaging Tests for Suspected Coronary Heart Disease |
| Improving Outcomes and Care Experience Among Dual Eligible Members: the Role of Health System Factors |
| Inpatient Outcomes Associated with Regional Implementation of a Benzodiazepine-Sparing Alcohol withdrawal Orderset |
| Integration of Standardized Ovarian Cyst Risk Stratification System into Radiology Reports Estimates Risk and informs Follow-Up |
| Mental Health Service Demand in the Face of Covid-19 |
| New Clinical Decision Support intervention increased Safe Outpatient Management of Emergency Department Patients with Pulmonary Embolism |
| New risk estimates for colon cancer among persons with serrated colon polyps inform guidelines for timing of repeat colonoscopy |
| Opioid Safety Education Associated with Decreased Opioid Prescribing by Emergency Physicians |
| Outcomes Following interventions To Sustain Body Weight in Esophageal Cancer Patients Starting Preoperative Therapy |
| Outcomes of Door-To-Needle Times in Stroke Patients |
| Outpatient Mastectomy: Factors influencing Patient Selection and Predictors of Return to Care |
| Outreach with Fit Testing increases Detection of Polyps/CRC Among Younger African Americans (45-50 Years) |
| Patients with severe asymptomatic carotid stenosis are at low risk of stroke with contemporary medical management |
| Rapid Case Ascertainment Using NLP Is Effective and Feasible in Pancreatic Cancer Management |
| Regionalization of Sub-Specialized Gastric Cancer Care increased Use of Laparoscopic Approaches, Recommended Staging, and increased Survival |
| Regionalization of Testicular Cancer Diagnosis and Treatment Planning Effective and increased Satisfaction Among Oncologists |
| Regionalizing Sub-Specialized Adrenal Surgery Decreases Operative Time, Hospital Stay, and Major Complications |
| Short-course treatment (8 weeks) as effective as 12 weeks treatment for black patients with hepatitis C virus (HCV) infection |
| Streamlined surgical and perioperative-care benefit esophageal cancer patients undergoing esophagectomy |
| Streamlining Genetic Counseling increases Genetic Testing Among Women with Ovarian Cancer |
| Structured reporting of lung nodules detected on chest CT was associated with greater chance of detecting early stage lung cancer |
| Systemic Identification and Management of Familial Hypercholesterolemia Optimizes Patient Recognition and Treatment |
| Telemedicine (Compared to in-Person Gastroenterology Visits) Had High Patient Satisfaction and Comparable Physician Decision-Making |
| Telepsychiatry Provides Rapid Mental Health Evaluation and Referral for Treatment Among Adults with Mild-to-Moderate Symptoms |
| The incidence of Venous Thromboembolism Is Similar in Outpatients with and without Sars-Cov-2 infection |
